# Phonological awareness training and phonological therapy approaches for specific language impairment children with speech sound disorders: a comparative outcome study

**DOI:** 10.1007/s00405-023-08274-5

**Published:** 2023-11-09

**Authors:** Heba Mahmoud Farag, Hossam Eldessouky, Elham Shahin, Mai Atef

**Affiliations:** ENT Department, Faculty of Medicine, Phoniatrics, Phoniatric Unit, Cairo University, King Faisal Street, 300, Cairo, Giza, 12511 Egypt

**Keywords:** Specific language impairment, Speech sound disorders, Phonological awareness training, Phonological therapy

## Abstract

**Purpose:**

Children with specific language impairment (SLI) might present with speech sound disorder (SSD) and phonological awareness (PA) deficits which put them at risk of potential reading problems. This work aimed to organize an intervention program in Arabic for phonological training and to assess the effect of PA training versus the phonological therapy (PT) for children with SLI and SSD.

**Methods:**

The study was carried out on 60 children with comorbid SLI and SSD, aged 5–7 years. Children were equally divided into two groups; each group received language therapy combined with (PT or PA training). Measures of language development, phonological output, and PA were taken before therapy and at 4 month post-therapy for all children.

**Results:**

The two therapy groups made nearly the same amount of progress in the development of language and phonological production, with no significant differences regarding language age and percent of consonants correct (PCC). The PA training group progressed more on the PA skills than children who received PT over the same time.

**Conclusions:**

PA training could facilitate the development of phonological skills by targeting the child’s awareness of phonemes and improving the production of sound patterns.

## Introduction

Specific language impairment (SLI) is characterized by persistent language delay that affects the everyday communication of children in the absence of a medical condition that could account for these symptoms [1]. SLI is a heterogeneous deficit; children with SLI can struggle with various language components, such as lexicon, syntax, morphology, and phonology [2].

Children with SLI may have deficits in sentence-level semantics; some authors found that SLI children did not include lists of subjects or objects nor plural responses for WH-questions [3]. They may also show lexical retrieval or pragmatic deficits [4]. Van der Lely [5] suggested a grammatical subtype of SLI, which includes phonological and syntactic deficits. A further dissociation between syntax and phonology in SLI was presented by Ebbels et al. [6].

The subgroup of SLI children with syntactic structure difficulties may become unnoticed as they include subtle elements that are difficult to be identified [7, 8]. However, SLI with speech sound difficulties is usually noticeable [9]. Children with SLI, when compared to their peers without difficulties, might present with speech sound disorder (SSD) [10, 11]. Some studies specifically concerned with the association between language impairment and SSD, Broomfield and Dodd [12] found robust bidirectional comorbidity between SSD and language impairment.

Speech sound disorder (SSD) is a broad term referring to any difficulty or combination of difficulties with articulation or phonological representation of speech sounds that lead to reduced speech intelligibility [13]. Phonological disorder (PD) occurs when a child's speech sound system is organized differently than his peers, with no apparent physiological etiology [14]. Signs and symptoms of SSD may occur as articulation deficits that focus on errors in producing individual speech sounds (e.g., distortions and substitutions), or as phonological deficits that focus on rule-based errors that affect more than one sound (e.g., fronting and final consonant deletion). Many researchers prefer to use the broader term “speech sound disorder”, when referring to speech errors of unknown reason [15, 16]. There are few reports regarding the comorbidity of SSD with language impairment, according to Shriberg et al. [17], the rate of comorbidity between SSD and language impairment in 6-year-old children was 1.3%, they found that approximately 5–8% of children with persisting SLI had speech delay.

Children with phonologically based SSD may be associated with poor phonological awareness; therefore, they are at risk for later literacy problems [18]. Thus, working on expressive phonological skills and phonological awareness is essential to support the underlying skills for literacy in children with SSD [19].

Several studies have investigated the effects of different intervention approaches on phonological deficits in children with SSD. Articulation-based therapy is one of the common treatments used to improve speech intelligibility in SSD, focusing on articulating individual phonemes “sounds” in speech. Over time, the focus of the intervention for SSD has been changed to the phonologically based therapy that focuses on “phonological rules”. Researchers demonstrated that the children who received phonological therapy showed greater generalization of the phonological rules in their speech than those who received articulation therapy [20].

The goal of phonological therapy (PT) is to suppress error patterns “phonological processes”, which are systematic sound changes that children adapt to simplify speech [21]. Various phonological processes could be identified in children with SSD, including syllable structure, substitution, and/or assimilation processes. Within these overall classifications, many specific phonological process deviations exist, such as cluster reduction, final consonant deletion, stopping, fronting, and labial or alveolar assimilation [22]. Children with SSD have been found to use some of these phonological processes in the Arabic language (e.g., /baћr/ (sea) is pronounced as /ba/, /serir/ (bed) is pronounced as /terir/ and /lamba/ (lamp) is pronounced as /mamba/).^1^

Different phonological approaches were documented for treatment of children with SSD [23]. The minimal pair approach [24] and cycles approach [25] are examples of the recognized phonological therapies. The minimal pair treatment aimed to introduce new phonemic distinctions in language through the use of pairs of words that differ by a single phoneme. The feature differences between the phonemes are the focus of treatment (e.g., the phonemes /*k*/ and /*t*/ differ in terms of place, back versus frontal. If the place distinction is learned in treatment of /*k*/–/*t*/ pairs, this same back frontal contrast will be carried over to other pairs, such as /*ɡ*/–/*d*/). The cycle’s phonological approach is one of the most common methods to treat children using many different phonological processes. It intensively targets one primary pattern (such as final consonants, clusters, velars, and liquids) for a fixed time, then the next primary pattern, and so on, until all primary patterns have been targeted, completing one cycle. Then, the second cycle begins, starting again with the first pattern but with more complicated targets. It aims to increase a child’s intelligibility by facilitating the emergence of the primary target patterns. Cycles are used to stimulate the emergence of a specific sound or pattern.

The information on the effectiveness of phonological awareness (PA) for children with SSD and its role in remediation is growing [26]. Gillon [19, 27] demonstrated that children with a phonologically based SSD benefitted from PA intervention. The PA therapy in Gillon's studies focused on developing PA at the phoneme level. PA intervention aims to facilitate change in phonological skills by targeting the child's awareness of the contrastive nature of sounds.

PA training has been a welcome addition to the rehabilitation of PD, and clinical research results have shown positive effects of PA intervention for PD in general [28]. The studies in Arabic on phonological awareness therapy are few, specifically its efficiency compared to other treatment approaches for phonologically based SSD. Different interventions should be evaluated and applied efficiently to children with SSD to investigate if they will benefit from them. This study aimed to plan a phonological awareness intervention program for Arabic-speaking SLI children with SSD, and to assess its effectiveness compared to the phonological therapy approach. It was important to know more about the effective intervention approach for those children through assessing specific outcomes (improvements in the PCC and enhanced PA skills).

## Materials and methods

The current study was conducted on 60 Egyptian Arabic-speaking children, from a similar medium socioeconomic background who were seeking medical advice at the phoniatrics unit, Kasr El-Aini hospital. Their age ranged from 5 to 7 years (mean = 68.50 ± 5.59 months), and there were 36 males (60%) and 24 females (40%). All children were diagnosed to have comorbid SLI and SSD. The children's selection inclusion criteria were impaired language and phonological skills more than one standard deviation below the mean expected for their age after assessment by a phoniatrician, average non-verbal IQ (greater than 85), and normal hearing, as shown by their hearing test. All children lacked the exclusionary criteria, such as any apparent neurological, motor, sensory, or behavioral disabilities that could cause language deficits. Children were equally divided into two groups (groups A and B) to compare further the effectiveness of each of the two different therapy approaches. Parental informed consent was obtained for all patients, and we adhered to the principles outlined in the Declaration of Helsinki. The protocol of the study was approved by the research ethics committee of faculty of medicine Cairo University N-143-2022.

### Assessment

We carried out the parents’ interview, and asked them about the child’s age, complaint, history of any perinatal disorders, developmental history, history of any childhood illness, and social behavior of the child. All children were subjected to a thorough clinical examination, including general, neurological, local ear, nose, and throat examinations. The examination revealed no abnormalities, and the children showed no apparent neurological, motor, sensory or behavioral deficits.

Stanford–Binet Intelligence Scale, ed 4, was used for all children to assess their IQ [29]. The Arabic version of the modified preschool language scale (PLS4) was used to evaluate language development in children under study [30]. This test can be used to detect receptive, expressive, and total language age. The percent of consonants correct (PCC) was calculated for all children to assess the speech sound disorders in SLI children [31]. The total number of correctly produced consonants was divided by the total number of target consonants in a list of words and multiplied by 100.

The Arabic phonological awareness assessment battery was used for all children in this study to assess their PA skills. This battery was replicated from the meta-phonological abilities battery after adapting the test items to the Arabic language [32]. The evaluation of PA in this study included the *segmentation and blending test*. Five words were presented to the child for segmentation assessment, and the child was asked to segment the word into syllables (e.g., the child was asked to segment the word /ko:ra/ into its component syllables as the following: /ko:/ and /ra/). Five words were presented to the child for blending assessment, and the child was asked to blend syllables into the word (e.g., the examiner presented the component syllables of the word to the child /do:/ and /Læb/, then the child was asked to combine the two syllables together to recognize the word /do:Læb/). *Phoneme matching test:* the child was asked to find the picture of a word that starts with the same sound as the spoken word, ten words were presented to the child (e.g., when presenting the word /ʃaʔr/ “hair”, the child was asked to choose the word that starts with the same sound with the target word, which is the word /ʃara:b/ “socks”). *Initial phoneme deletion test*: the child was asked to identify the picture of a word that is formed after the deletion of the initial consonant of the spoken word, ten words were presented to the child (e.g., when presenting the word /ћfa:r/ “digger”, the child was asked to isolate the first sound heard in the word, which is the /ћ/ sound and say the word /fa:r/ “mouse”). *Rhyme matching test:* the child was asked to identify which one of the three test word items rhymes with the spoken word produced by the examiner (e.g., when presenting the word /bæ:b/ “door”, the child was also presented with the words /bata/, /kæ:b/ & /tæg/. The child was then asked to choose the word that would rhyme with the target word /bæ:b/, which is / kæ:b/), ten words were presented to the child for rhyme assessment. The child would get a score of “1” for each correct response, and the total score of each one of the above four tests was 10.

### Intervention

The children were randomly assigned to one of two treatment groups. Each child received four sessions per week for 4 months, lasting about 30 min. Two sessions per week included language intervention**,** and two sessions focused either on phonological therapy (Group A) or phonological awareness training (Group B). The intervention was performed by a phoniatrician in the phoniatric unit of Kasr El-Aini hospital.

The phonological therapy in this study was based on the cycle’s phonological approach [33], which targeted the error patterns. Once the target is determined (e.g., if /*ɡ*/ produced as /*d*/ and /*k*/ produced as /*t*/ indicating the same error pattern, i.e., fronting), the cycle begins with one primary error pattern (e.g., when working on fronting, the target is the production of velar sounds, we choose five familiar words with /*k*/ and use focused auditory bombardment with lots of practice within a cycle every 1 to 2 weeks). We move from one primary pattern to the next one, whether or not the child has corrected the first pattern (e.g., when working on stopping, the target is the production of fricatives, words with sounds /*s*/, /*f*/ will be used for training), until all the primary error patterns have been targeted, this completes one cycle. Another cycle begins, targeting one or more different phonological patterns. There is no predetermined level of mastery of phonemes within each cycle. Cycles are used to stimulate the emergence of a specific sound or pattern, not to produce mastery. Recycling of phonological patterns continues until the targeted patterns are present in the child’s spontaneity. The goal is to approximate the gradual typical phonological development of SLI children with phonologically based SSD.

The PA training program in this study was applied from a training program adapted for Arabic-speaking children [34]. Phonological awareness training included: *segmentation of syllables:* by training the child to segment the word into syllables and repeat it many times (e.g., train the child to segment the word /Læbæn/ “milk” into /læ/ and /bæn/). We may need to clap with segmentation and ask the child to segment the words. *Blending syllables:* by training the child to recognize a word presented by the investigator as separate syllables (e.g., for the word /sæʔæ/ “watch”, the investigator presents separately, the component syllables of the target word as follows: /sæ/ + /ʔæ/ and the child is trained to combine the isolated syllables together to say the target word).

*Initial phonemes matching:* by training the child to recognize picture pairs match in the same initial sound. *Initial phoneme deletion:* by training the child to find out what the remaining word will be (choose from the pictures) if we delete the first phoneme of the word (e.g., when presenting the word /fna:r/ “lighthouse”, the child is instructed to isolate the first sound heard in the word, which is the /f/ sound and say the word /na:r/ “fire”). *Rhyming detection:* by training the child to recognize the two rhyming words from the three words presented to him (e.g., the child is trained to choose words /kæ:s/ and /ræ:s/ that rhyme together from the three words: /kæ:s/, /bæ:b/ and /ræ:s/).

The children of groups A and B received pre- and post-therapy assessments to compare language age, PCC, and PA skills before and after the intervention. Pre-tests were taken immediately before the intervention, and the children did not receive the intervention before this study. Post-tests were carried out in a follow-up after 4 months of the intervention.

#### Evidence of content validity

Three independent and experienced phoniatricians judged all items of the language and phonological assessment and rehabilitation batteries for being completely relevant to the purpose for which they were meant. A high degree of validity was obtained in this study by the high agreement among evaluators.

### Statistical analysis

The statistical package of social sciences (SPSS) (version 28) was used to analyze the results. The normality of the data was tested using the Kolmogorov–Smirnov test. Numerical variables were presented as mean and standard deviation (SD). As the data were not normally distributed, the comparison between groups was conducted using the Mann–Whitney test, while the comparison between pre- and post-treatment was performed using Wilcoxon Signed Rank Test, with Bonferroni correction of *p* value carried out to avoid hyperinflation of type-1 error due to multiple comparisons. A (*P* ≤ 0.05) was considered significant.

## Results

There was no significant difference between both groups A and B regarding receptive, expressive and total language ages before therapy. Children within group A showed significant improvement in the receptive, expressive and total language ages after language and phonological therapy. Children within group B showed significant improvement in the receptive, expressive and total language ages after language and phonological awareness training. There was no significant difference between both groups A and B regarding receptive, expressive and total language ages after therapy (Table 1).

**Table 1:** Comparison within and between group A and group B regarding chronological and language ages before therapy and after therapy

	Group A Mean ± SD	Group B Mean ± SD	Test statistics	Effect size	*p* value (between groups)
Chronological age pre-therapy	68.13 ± 5.80	68.50 ± 5.59			0.500
Receptive language age pre-therapy	51.1 ± 10.5	51.1 ± 10.7	435	0.057	1.00
Receptive language age post-therapy	54.50 ± 10.6	53.9 ± 10.9	399.5	0.194	0.589
Test statistics	465	435			
*p* value (within groups)	< 0.001	< 0.001			
Adjusted *p* value	< 0.001*	< 0.001*			
Expressive language age pre-therapy	47.1 ± 9.4	47.1 ± 9.6	438	0.046	0.963
Expressive language age post-therapy	49.8 ± 0.5	49.6 ± 9.7	432.5	0.067	0.970
Test statistics	465	435			
*p *value (within groups)	< 0.001	< 0.001			
Adjusted *p* value	< 0.001*	< 0.001*			
Total language age pre-therapy	49.1 ± 9.7	49.0 ± 9.8	434.5	0.059	0.994
Total language age post-therapy	52.1 ± 9.6	51.4 ± 9.8	413.5	0.14	0.774
Test statistics	465	406			
*p* value (within groups)	< 0.001	< 0.001			
Adjusted *p* value	< 0.001*	< 0.001*			

*SD* standard deviation

Group A = Phonological therapy

Group B = Phonological awareness training

Analysis was done using Mann–Whitney test between groups and using Wilcoxon Signed Rank Test between pre- and post-treatment, with Bonferroni correction of *p* value done to avoid hyperinflation of type 1 error due to multiple comparisons

*Significant *P* value ≤ 0.05

There was no significant difference between both groups A and B regarding the PCC and PA scores before therapy. Children within group A showed significant improvement in PCC and PA scores after therapy. Children within group B showed significant improvement in PCC and PA scores after therapy. There was no significant difference between both groups A and B regarding the improvement of PCC after therapy. Children within group B showed significant improvement in all phonological awareness scores than children within group A after therapy (Table 2).

**Table 2:** Comparison within and between group A and group B before therapy and after therapy regarding percent of consonant correct (PCC) and phonological awareness scores

	Group A Mean ± SD	Group B Mean ± SD	Test statistics	Effect size	*p* value (between groups)	Adjusted *p* value
Segmentation and blending pre-therapy	1.17 ± 1.02	1.6 ± 1.4	491	0.157	0.380	
Segmentation and blending post-therapy	2.7 ± 1.4	4.9 ± 1.8	722	1.215	< 0.001	< 0.001*
Test statistics	351	406				
*p* value (within groups)	< 0.001	< 0.001				
Adjusted *p* value	< 0.001*	< 0.001*				
Phoneme matching test pre-therapy	1.17 ± 1.1	1.4 ± 1.3	472.5	0.086	0.554	
Phoneme matching test post therapy	2.3 ± 1.1	5.1 ± 1.7	793.5	1.737	< 0.001	< 0.001*
Test statistics	210	435				
*p* value (within groups)	< 0.001	< 0.001				
Adjusted *p* value	< 0.001*	< 0.001*				
Initial phoneme detection pre-therapy	0.77 ± 0.73	.97 ± .78	496	0.176	0.321	
Initial phoneme detection post-therapy	1.9 ± .8	2.5 ± .574	610.5	0.644	0.004	0.016*
Test statistics	300	378				
*p* value (within groups)	< 0.001	< 0.001				
Adjusted *p* value	< 0.001*	< 0.001*				
Rhyme matching test pre-therapy	0.93 ± 1.08	1.1 ± 1.4	450.5	0.002	0.800	
Rhyme matching test post-therapy	1.8 ± 1.3	4.5 ± 1.4	790	1.706	< 0.001	< 0.001*
Test statistics	253	435				
*p* value (within groups)	< 0.001	< 0.001				
Adjusted *p* value	< 0.001*	< 0.001*				
PCC pre-therapy	75.2 ± 3.1	75.2 ± 3.1	433	0.065	0.976	
PCC post-therapy	83.6 ± 4.3	81.8 ± 3.9	307	0.567	0.052	0.208
Test statistics	432	435				
*p* value (within groups)	< 0.001	< 0.001				
Adjusted *p* value	< 0.001*	< 0.001*				

*SD* standard deviation

Group A = Phonological therapy

Group B = Phonological awareness training

Analysis was done using Mann–Whitney test between groups and using Wilcoxon Signed Rank Test between pre- and post-treatment, with Bonferroni correction of *p* value done to avoid hyperinflation of type 1 error due to multiple comparisons

*Significant *P* value ≤ 0.05

## Discussion

Children with SLI and SSD are at risk for literacy difficulty, which can be attributed to their language and phonological problems. Some studies have traditionally focused on phonological/language-based treatment approaches for children with SSD [26]. Phonological awareness intervention for children with SSD has been recommended; however, not much has been published regarding the efficacy of PA training in Arabic. This research was carried out to present and assess the effect of PA training in contrast with phonological therapy in treating SSD in children with SLI.

Each of the two therapy approaches, either phonological therapy or phonological awareness training, was presented to children in this study in addition to the direct language therapy. The results showed significant improvement after therapy for groups A and B regarding receptive, expressive, and total language ages, as shown in Table 1. This indicated that direct language therapy facilitated various language aspects of SLI children. It helped children to find the right words and to form more complex language structures, particularly those who manifest syntactic difficulties. Our result goes in line with the study of Munro et al. [35], which showed a significant improvement in different language measures after individual language intervention sessions for SLI children.

In our study, there was no significant difference between both groups A and B regarding receptive, expressive, and total language scores after therapy, as shown in Table 1. These results could be explained by the fact that the DLD children of both groups promoted equal improvements in language domains, because they received the same number and quality of sessions of direct language therapy.

The children within group A showed significant improvement in PCC scores after therapy (Table 2). This indicated that children with SSD could learn several sounds that were being worked on simultaneously during phonological therapy. Our study’s basic concern of phonological therapy was the phonological rules as cycles were used to stimulate the emergence of specific sounds or patterns. This result demonstrated that the cycle phonological approach as a phonologically based approach could decrease phonological errors in children with SSD. Our result coincides with the results of other studies by Mota and Pereira [36] and Mota et al. [37], who have reported gains in phonological outcomes for children who undergo the cycles approach. Moreover, Blanco [38] studied a group of children with PD, treated by cycles approach, and observed children’s improvement regarding the acquired phonemes in their phonological systems.

The scores of all phonological awareness tests were significantly improved within group A after therapy (Table 2), though this group did not receive direct phonological awareness training. These results indicated that a relationship would be found between production phonology and phonological awareness. Thus, the correction of phonological errors resulted in the improvement of PA skills. The improved phonological system of the treated group facilitated the development of their poor phonological awareness skills. In addition, the study of Preston and Edwards [39] suggested the relationship between phonological errors and poor phonological awareness skills, because both reflect weak phonological representations. Their study found that atypical sound changes predicted about 13% of the variance in PA and demonstrated that the children who produced more atypical sound changes performed more poorly on the PA tasks.

The children within group B showed significant improvement in PCC scores after therapy, as shown in Table 2. This result elucidated that the phonological awareness intervention could facilitate change in the phonological skills by targeting the child’s awareness of the contrastive nature of sounds while also working on producing sound patterns. The PA training could allow the child to organize internal representations of sounds and to reflect on phonemes’ production. Our result goes with the study of Adams et al. [40], who found a significant improvement in PCC when they examined the effects of a program targeted at improving PA skills for children with PD. There have been several studies that have provided positive evidence to support phonological awareness intervention. Hesketh et al. [41] found that PA therapy was as effective as traditional therapies, which target the production of speech sounds more directly. In some studies, improvement in phonological awareness has been targeted to prepare the child for direct intervention on speech [42]. Other studies targeted improvement in phonological awareness skills, because improvement in speech will automatically follow [43]. However, Denne et al. [44] found that speech production results were less convincing than the significant progress on the phonological awareness measure made by the group who received phonological awareness treatment.

In our study, there was a significant improvement of all phonological awareness scores after therapy in children within group B. This is thought to be an unsurprising result, because phonological awareness therapy should have its strongest effect on the group who directly received PA training. Furthermore, the results suggested that children with SSD may need more therapy focusing on their phonological awareness skills to improve the development of the phonological system. All test items of PA were improved (segmentation and blending, phoneme matching, initial phoneme deletion, and rhyming). This improvement of PA is thought to be an important factor in improving reading abilities in those children per many meta-analyses studies that have provided evidence that phonological awareness training can facilitate the learning process [45]. Therefore, our results could recommend PA training for children with SLI and SSD before starting reading education.

A comparison between the effectiveness of the two therapy approaches in this study showed no significant difference concerning the PCC scores after therapy between groups A and B, as shown in Table 2. Our results showed that both approaches could effectively improve productive phonological skills for children with SLI and SSD. Although the two interventions had different selected targets and procedural differences, including different instructions and therapy activities, both of them helped the improvement in the PCC of the treated groups. The relatively equal effectiveness of both approaches on speech sound production could be explained by the fact that both models could improve the phonological system of children with SSD. This result agrees with Gillon’s study [46], which showed that PA therapy was as effective as the intervention approaches that targeted speech production and expressive language in improving the PCC and had significantly stronger effects on measures of phonological awareness. Other study by Gillon and Macfarlane [47], have also shown that PA training strategies were associated with significant improvements in children’s speech production.

In our study, a comparison between groups A and B regarding the results of phonological awareness after therapy demonstrated that children with group B made more significant progress on all phonological awareness skills than children who received phonological therapy. This indicated that the phonological awareness approach could allow more opportunities for developing phonological awareness skills in conjunction with improving speech sound production. The results of our study coincide with the study of Gillon [27] in which the children with spoken language impairment participated in either 20 h of PA intervention, 20 h of a control intervention targeting speech production, and expressive language, or minimal intervention focusing on speech production. The study reported that the children in the PA intervention group made advanced progress on phoneme awareness tasks reaching levels similar to typically developing children at the post-intervention assessment. The children who received the PA intervention showed significantly improved phonological awareness skills than those who received language-based speech intervention.

The beneficial effects of phonological awareness training for children with SSD were also observed by Adams et al. [40], who found that children’s phonological skills were improved over a relatively short period of meta-phonological therapy. However, Harbers et al. [48] were more cautious about their interpretation of the benefits of phonological awareness training for speech production performance. They found that the rate and degree of change in phonological awareness did not always parallel the production performance**.** Another view was declared by Stackhouse et al. [49], who studied the relationship between phonological awareness training and phonological therapy; they suggested that phonological awareness cannot be dealt with independently as it is an integral part of phonological intervention.

These results can suggest that it would be helpful to apply interventions focused on phonological awareness that could lead to gains in phonological production for Arabic-speaking children with SLI and SSD.

## Conclusions

This study can only be seen as a first step in investigating PA intervention's role in the improvement of the phonological production in Arabic-speaking children with SLI and SSD. We found that children with SLI and SSD showed improvement of their phonological production and they developed more phonological awareness skills concurrently when provided with phonological awareness intervention in contrast to a traditional phonological intervention. Our results could contribute to the literature by asserting the improvement in children’s phonological system with PA therapy application in children with language and speech difficulties. This study could be useful in highlighting the advantage of implementing phonological awareness training in future therapy approaches as a more holistic program for development of children phonological system with underlying phonological awareness skills crucial for academic performance, thus children’s risk of academic difficulties is to be reduced.

The absence of the control group in this study is considered as a limiting factor, and it should be taken into account that further studies will include the control group to ensure that the improvement is an effect of the intervention. In addition, further research will be needed to assess the stability of PA therapy effect and its later influence on reading. We hope this study will help to stimulate additional well-controlled studies for a larger group of children with SLI and SSD to validate the effect of training for remediation of these children.

## Data Availability

The data that support the findings of this study are available on request form the corresponding author. The data are not publicly available due to their containing information that could compromise the privacy of the research participants.

## Footnotes

1 International Phonetic Alphabet (IPA) was used for representation of speech sounds in written form.
